# Experimentation Management in the Co-Created Smart-City: Incentivization and Citizen Engagement †

**DOI:** 10.3390/s19020411

**Published:** 2019-01-20

**Authors:** Johnny Choque, Luis Diez, Arturo Medela, Luis Muñoz

**Affiliations:** 1 Communications Department, University of Cantabria, Santander 39005, Spain; jchoque@tlmat.unican.es (J.C.); luis@tlmat.unican.es (L.M.); 2 Tecnologías, Servicios Telemáticos y Sistemas S.A., Santander 39011, Spain; amedela@tst-sistemas.es

**Keywords:** smart city, experimentation, EaaS, co-creation, incentivization, rewarding, communities

## Abstract

Under the smart city paradigm, cities are changing at a rapid pace. In this context, it is necessary to develop tools that allow service providers to perform rapid deployments of novel solutions that can be validated by citizens. In this sense, the OrganiCity experimentation-as-a-service platform brings about a unique solution to experiment with new urban services in a co-creative way, among all the involved stakeholders. On top of this, it is also necessary to ensure that users are engaged in the experimentation process, so as to guarantee that the resulting services actually fulfill their needs. In this work, we present the engagement monitoring framework that has been developed within the OrganiCity platform. This framework permits the tailored definition of metrics according to the experiment characteristics and provides valuable information about how citizens react to service modifications and incentivization campaigns.

## 1. Introduction

Currently, information and communication technologies (ICT) are playing a key role in the evolution of cities to provide more efficient and sustainable services. In particular, Internet of Things (IoT) solutions allow the possibility of leveraging heterogeneous devices, in terms of capabilities or mobility, to gather service-related information, and to enforce actions, exploiting techniques such as machine learning or big data analysis. These aspects make ICT appear as the most suitable way to bring novel services to citizens. Moreover, the possibility to establish bi-directional communication between city authorities and citizens poses a change in the way services are provisioned, shifting from a bottom-up to a horizontal relationship.

This new scenario permits citizens to play a far more active role in all the stages of the services’ creation, embracing their design, development, and evolution. For this to happen, it is necessary to develop tools and frameworks that allow local authorities, and service managers, to define and develop services, along with their potential users, in a co-creative and agile manner, taking advantage of the facilities brought about by ICT.

The co-creation of services in general, and of urban services in particular, is believed to have important benefits for service providers. Traditionally, the participatory approach has been associated with the generation of ideas and the conception of new products. However, in ever-changing scenarios like cities, the co-creation of services can help to further evolve them, according to new needs and bounding conditions. In this sense, the service co-creation would allow a faster deployment of innovative urban solutions [1], since they are more likely to be easily adopted by citizens.

In the last few years, co-creation and ICT have been jointly applied to a large set of realms. Some works have applied co-creation to improve the quality of services, or to engage citizens by using gaming or augmented reality [2,3]. Others works, such as [4], aimed to increase people participation in the government by social media, taking advantage of the increasing concern about social aspects. Regarding the smart city paradigm, some works have fostered co-creation to solve specific problems in urban environments, like [5], where authors followed a strategy based on hackathons, or [6], where machine-to-machine communications was used. Finally, collaborative methodologies have also been used to bring service providers near citizens interested in the co-creation process [7].

Although all the aforementioned works address the objective of citizen engagement, they are limited to either some application areas or to solving one specific challenge. In addition, those solutions closer to the application of ICT to the smart city lack a means to assess the level of citizen engagement. Opposed to that, the OrganiCity (OC) project (https://organicity.eu/) addressed the definition and deployment of a holistic framework, capable of performing systematic urban experimentation in a co-creative manner. In addition, it implements functionalities to monitor the engagement degree, whose definition can be tailored according to the service needs. Precisely, this paper, which is an extension of that showcased in [8] and presents the citizen engagement methodology and monitoring mechanisms that have been developed within the experimentation management (EM) suite of OC.

Some prior works have presented methodologies to foster citizen participation in the development of the smart-city. For instance, in [9], the authors presented a methodology for citizens to evaluate urban services, that is a case study in a living lab as a micro city. Differently, the framework presented herein provides a means to define quantitative engagement metrics, that are specified by service developers. In this sense, this work does not provide a one-size-fits-all solution for citizen engagement, but a set of functionalities to define the best solution for each case. These functionalities were released as advanced services of the second open call of the OC project, in which 17 cross-city experiment groups developed novel urban services on top of the OC platform (https://organicity.eu/experiments/). Throughout this paper, accepted open call projects are termed experiments, which test an idea at a small scale through various methods (including data and technology) to tackle a specific urban challenge. To the best of our knowledge, this is the first development of a framework to define tailored quantitative engagement metrics for urban experimentation, and that is validated in the field at this scale.

The rest of this paper is organized as follows. In Section 2, we provide an overall description of the OrganiCity facility, highlighting its main services and components. Afterwards, Section 3 focuses on the main aspects of the experimentation management framework, where its three main entities are described. Then, in Section 4, we describe the engagement monitoring flow, pointing out the role played by the aforementioned entities and providing practical implementation details. In Section 5, we focus on the concept of the metric, which is the cornerstone of the quantitative engagement monitoring. Afterwards, the experimentation management in general, and the engagement functionalities in particular, is assessed in Section 6 by analyzing the usage of OC services during the second OC open call and how such an experimentation period helped to evolve the urban services. Finally, Section 7 concludes the paper, summarizing the work and describing the next steps to be tackled.

## 2. OrganiCity Facility Architecture

**Figure 1:**
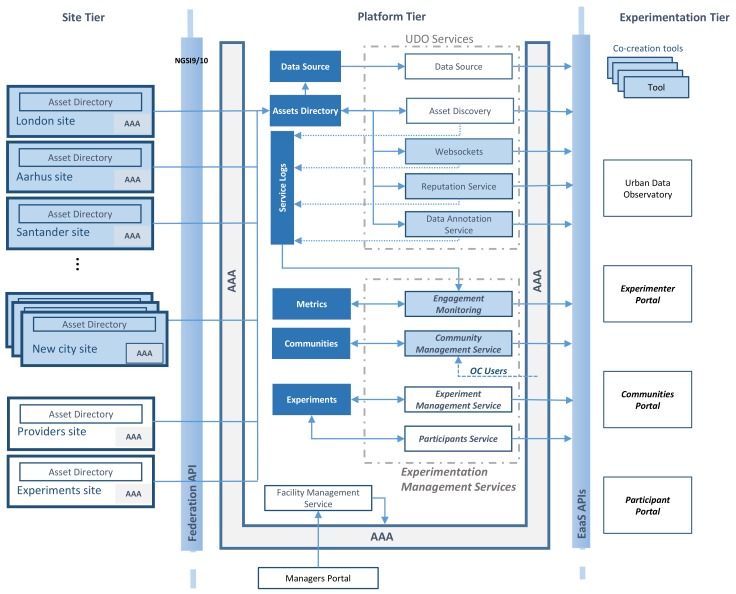
Overarching description of the OrganiCity EaaS facility.

One of the main outcomes of the OrganiCity project is the definition of a reference experimentation-as-a-service (EaaS) architecture and its implementation by means of the OC EaaS platform. The EaaS platform aims to bring a common ground where new concepts of urban services can be validated before moving to a production stage. In this sense, the platform can be utilized by local authorities, companies of different sizes, or even proactive citizens with innovative ideas. The reference architecture follows a tiered model [10], as depicted in Figure 1, which addresses data provisioning, platform management, and experimentation/service support and monitoring.

In particular, the first tier, namely the *Site Tier*, is made of entities providing datasets through a federation application programming interface (API), as depicted at the left of Figure 1, which can be afterwards exploited by the experimenting services. It is worth noting that the architecture does not pose any restriction to data providers, who can range from cities to private companies, each with their defined privacy level. In addition, data created during the experimentation are fed back to the platform, so that they can be used either by the experiment that created them or by others.

Afterwards, the *Platform Tier* is responsible for performing all the management tasks, including authentication, authorization, and accounting (AAA) policies. This tier presents the federated data to experimenters according to access policies and permits the creation of new data. In addition, the *Platform Tier* exposes a set of EaaS APIs that are used to provide experimentation services. Finally, experimenters interact with the platform through the *Experimentation Tier*, which brings a set of services and tools to ease the development of prototypical services and its management, both through programmatic APIs or by means of graphical interfaces. In a nutshell, we can divide services offered by the *Experimentation Tier* into those related to data, the ones responsible for managing the experimentation flow, and co-creation tools.

The data-related services allow discovering, exploring, and using both urban IoT and social data streams to build new solutions within cities. Furthermore, these services provide mechanisms to enrich data assets with annotations from users or even comments, as well as data reputation. Through the *Experimentation Tier*, these services are individually exposed through their corresponding APIs. In addition, all of them are integrated within the Urban Data Observatory (UDO), which is an extensible web portal featuring a large number of urban data-related functionalities.

In a similar way, the experimentation support services are also exposed both through APIs and web portals. The following sections will be devoted to thoroughly describe these services, paying special attention to the engagement monitoring functionality.

Finally, the *Experimentation Tier* brings services intended to simplify the development of new urban services, the so-called co-creation tools. It is worth noting that these co-creation tools have been conceived of to cover the needs of both technical and non-technical experimenters, so that they have been designed to be easy-to-use. In particular, the co-creation tools focus on the definition of urban scenarios, simple development of mobile applications, and integration of new hardware devices.

## 3. Experimentation Management Ecosystem

One of the key aspects of the OC platform is the implementation of an EM system, able to fulfill the requirements of potentially heterogeneous experiments. This system has been implemented as a set of APIs exposed through three web portals, which have been designed based on the three entities of the methodology described in Section 3: experiment, community, and participant. The key entity of the experimentation management framework is the experiment, while the others are linked to it to obtain or provide information to carry out the orchestration of engaging mechanisms. As commented on before, the EM framework lies in the *Experimentation Tier*, and it is used to enable the interaction of the application, and services belonging to experiments, with the platform.

Before describing the entities of the EM framework, it is necessary to describe the roles that different users may play within OrganiCity; the reader may refer to [10,11] for further information. In a nutshell, any user needs to create a user account, which includes user profile information, thus becoming an OC user. Afterwards, the user can obtain one or more of the following roles:

- Platform managers: They are responsible for controlling the correct operation of the OrganiCity platform as a whole, as well as the federation of sites and data storage.
- Site managers: For each site, its managers supervise how the site data are federated. This embraces data feeding and updating, as well as options on the data storage. This role is granted by the platform managers.
- Experimenters: These are people who want to test novel applications or services under the scope of an experiment. Thus, experimenters are allowed to control all the aspects of their experiments. In order to keep control of the platform resources, this role is granted by the platform managers.
- Participants: Finally, participants are people registered within OrganiCity who are willing to take part in experimenters as service users. In this sense, they test applications, and provide feedback to help in developing novel services in a co-creative way. Differently from the aforementioned roles, any OC user may decide to have a participant role.

### 3.1. Experiment Entity

An experiment is defined as a virtual testbed that enables a systematic experimentation. This involves experimenting in a methodological manner, evaluating the results of each step to inform the development team of the next steps or to modify the experiment settings. In this sense, the methodology modeled around the experiment entity enables enhancing the technology maturity of the experiments, as we will describe in Section 6.

The main functionalities created on the basis of the experiment entity are related to its management and also cover the deployment and experimentation process:

- Creation and management of experiments: An experiment can have different levels of complexity. As minimal requirements, an experiment only needs a name and a description. In addition, experimenters may define geographical regions where it takes place and advanced mechanisms to measure the user engagement over such regions [12].
  Furthermore, experimenters can define privacy levels for both the experiment itself and the data created within it. When data are defined as private, they can be only consumed by applications belonging to the experiment, which are identified by using the appropriate credentials provided by the AAA functionalities. On the other hand, when the experiment itself is defined as private, it is not visible to participants, so that they cannot freely join the experiment. Finally, it is worth noting that, at any moment, experiments can be stopped/restarted and edited (e.g., name or description).
- Creation and management of data assets: The experimenters can use a graphical user-interface to create and edit data assets, which helps them become familiar with the required format (NGSIcontext information model [13]). Besides, using the the appropriate credentials, experimenters can also create, edit, and delete data assets in a programmatic way through the corresponding APIs; see [10].
- Experiment team management: Depending on the size of the experiment, it may be necessary for several people to manage it. In this sense, an experiment team is a group of experimenters designated by the owner, who are allowed to manage the experiment. It is worth noting that the experiment owner can edit the team at any moment of its lifetime, by including or excluding members.

Finally, experimenters can define participation metrics in order to measure the level of engagement of participants. Later on, these metrics can be used as engagement indicators, so that incentivization measures can be applied accordingly. Metrics’ definition will be elaborated in detail in Section 5. In addition, it is worth highlighting that experiments are not associated with any city (i.e., site), so that it is possible to create cross-city experiments.

All the aforementioned functionalities are implemented in the experimenter portal (EP) (https://experimenters.organicity.eu), which in turn is the entry point of experimenters to the OC platform and serves as a hub for the experimentation services. Following the micro-service design of the entire OC platform, the EP incorporates services as independent views, which may eventually be deactivated depending on the actual requirements.

### 3.2. Community Entity

A community is a virtual group of users, and it is used by its creator to perform actions over such groups, instead of individually. In this sense, the objective of defining a community differs according to the role that creates it.

Platform and site managers have global scope, so that their communities apply filters over the entire list of registered users, irrespective of their roles. These communities are used to communicate with OC users, for instance in case of technical maintenance.

On the other side, communities defined by experimenters can be only made out of participants. This way, experimenters can group users with some particular interest or preference (according to the information provided in their profiles), or define communities with those participants that were active in the past. Afterwards, experimenters can invite users of their communities to enroll in the experiments or send notifications of promotions and marketing campaigns, according to some strategy of incentives and rewards. Besides that, it is also possible to define communities involving participants of different experiments to promote the relationship between them, to define community templates that help experimenters to create their own groups, or even create communities that promote user participation in activities carried out at the entire project level.

Furthermore, experimenters and managers are not aware of the actual identity of the users in their communities, but they only see indicators and the characteristics of their community’s audience. In a similar way, OC users do not know which communities they belong to, since such virtual groups are intended for managers and experimenters. In addition, being a community member does not pose any constraint on participants. Conversely, they can be a member of different communities.

Like experiment-related functionalities, those defined for communities are implemented in the communities portal (CP) (https://communities.organicity.eu), a web application that provides platform and site managers, as well as experimenters, user-friendly tools to manage the communities.

### 3.3. Participant Entity

This entity represents the engaged citizens and stakeholders (data scientists, city decision makers, organizations, etc.) that actively contribute to the creation of the OC facility tools and, on the other hand, become part of the co-creation experiments for future smart city making. In the context of OC, the participant are users registered in OC, who have stated their willingness to participate in experiments and decide on the experiments in which they take part. Similar to the aforementioned entities, the web application participant portal (PP) (https://participants.organicity.eu) implements the functionalities defined for the participant entity, which are the following:

- Experiment search: All public experiments are visible for participants, so that they can proactively decide to enroll in those they prefer, based on the experiment description. For instance, participants can chose experiments active in their city or those aligned with their preferences.
- Notifications: Participants can be notified about events happening in the experiments they are involved in, and they can be invited to new ones. It is worth noting that all interactions happen in an anonymous way, so experimenters are not aware of the identity of participants.
- Enrolling management: Participants can decide to leave an experiment at any point. Thus is applied to both experiments they proactively joined and those to which they were invited.

## 4. Citizen Engagement Methodology

**Figure 2:**
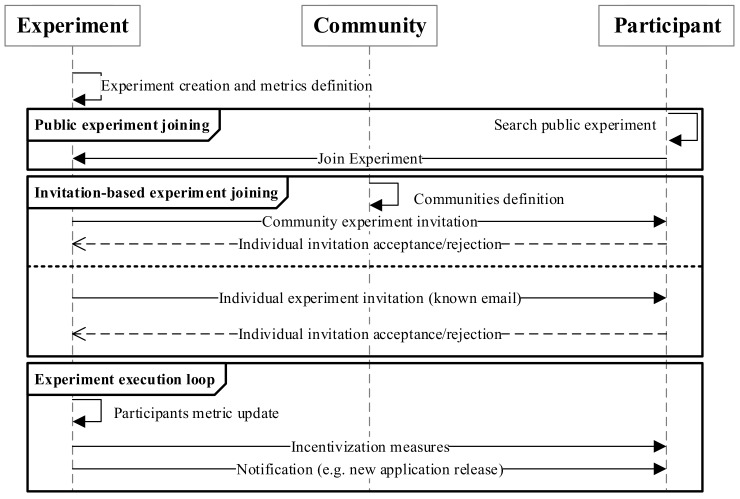
Overall OrganiCity experimentation flow.

The OrganiCity experimentation management system provides a palette of functionalities that cope with the main requirements to perform systematic experimentation by means of a clear experimentation flow, as shown in Figure 2.

First, using the tools provided by EP, experiments are created along with metrics to monitor the participants’ engagement level. At this point, the experimenter may decide whether to declare the experiment as public or private. In the case of public experiments, participants can freely join them, looking for them with the tools offered in the PP. Otherwise, experimenters must send bulk invitations using the previously-defined communities or individually to already known people. Eventually, participants can start using experiments, leading to updates in the engagement metrics. This way, experimenters can monitor how potential users react to their applications and services and take measures to further engage them. As can be observed, the whole experimentation is no longer seen as a linear process, but as a cyclic one where interactions between experimenters (i.e., service providers) and users (participants) evolve.

As can be seen in Figure 2, the key entity of this methodology is the experiment, while the other ones are linked to enable the orchestration of engaging mechanisms. To better understand the citizen engagement methodology and the role that the aforementioned entities play in it, in the following, we describe how they are related from a practical perspective.

**Figure 3:**
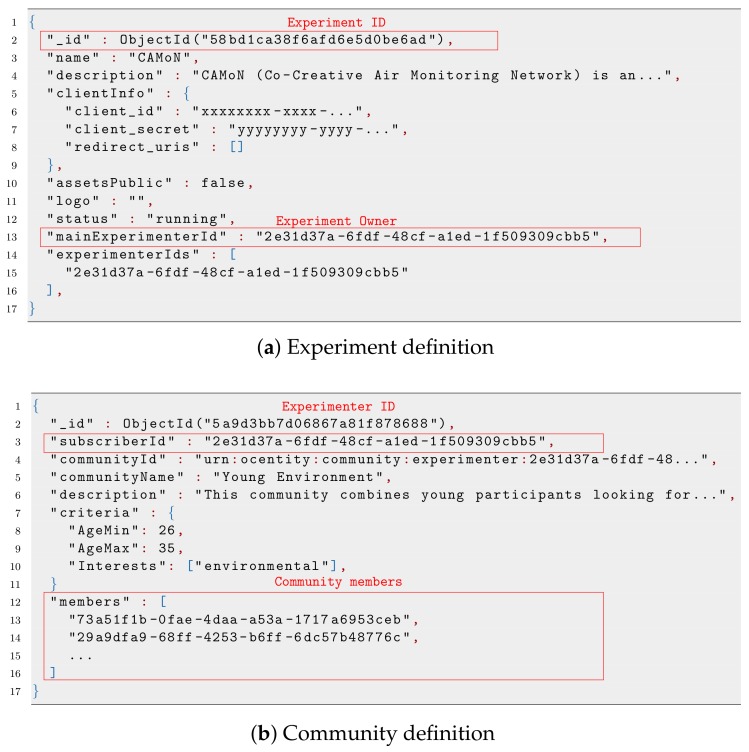
Illustrative example of experiment and communities defined during an experiment. In both cases, relevant identifiers are highlighted.

Figure 3a shows the JavaScript Object Notation (JSON) file that represents a basic definition of an experiment, where we highlight the two pieces of information that permit linking other components with it. The former is the *_id*, which is used as a unique identifier of the experiment. Other components use it to reference the experiment or to define their dependency on it. The latter is *mainExperimenterId*, which is the identifier of the experiment owner, who has total control over it.

The other entity that has a close relationship with the experiment is the community, whose information is represented in Figure 3b. As can be observed, during the creation of a community, the system takes the experimenter’s identifier, indicated by the *subscriberId* key, to set the ownership of the community. In addition, the list of OC users belonging to the community is defined by the *members* key, which includes their corresponding identifiers defined during the registration in the OC platform.

**Figure 4:**
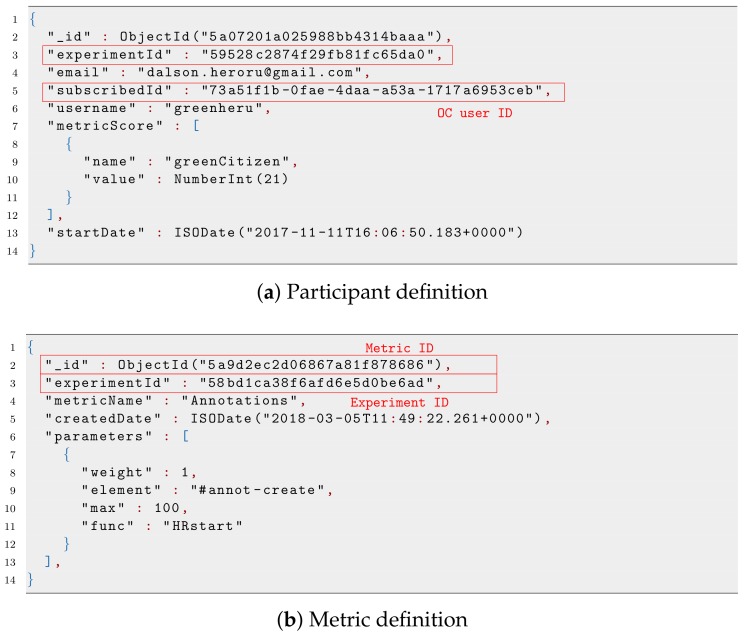
Examples of participant and metrics defined for one experiment.

Finally, the participant entity is showcased in Figure 4a. Following the same pattern described before, this entity holds the identifier of the concerned OC user in the *subscriberId* key, as well as the identifier of the experiment for which it is a participant. As can be observed, the last values of the metrics defined for the experiment are also stored, while the evolution of the metric over time for that participant is separately stored, as we will see later.

As for the metrics, there exists another entity in which its definition is stored, as depicted in Figure 4b. This entity is uniquely identified by the *_id* key and linked to the experiment under which such metric was defined by the key *experimentId*.

**Figure 5:**
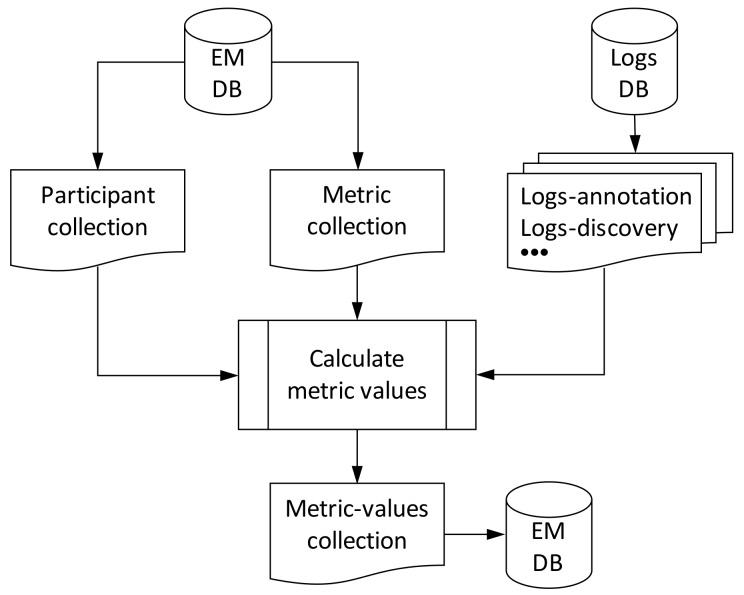
Description of the logging system. Currently the annotations, discovery services provide the system with the logs to define metrics.

**Figure 6:**
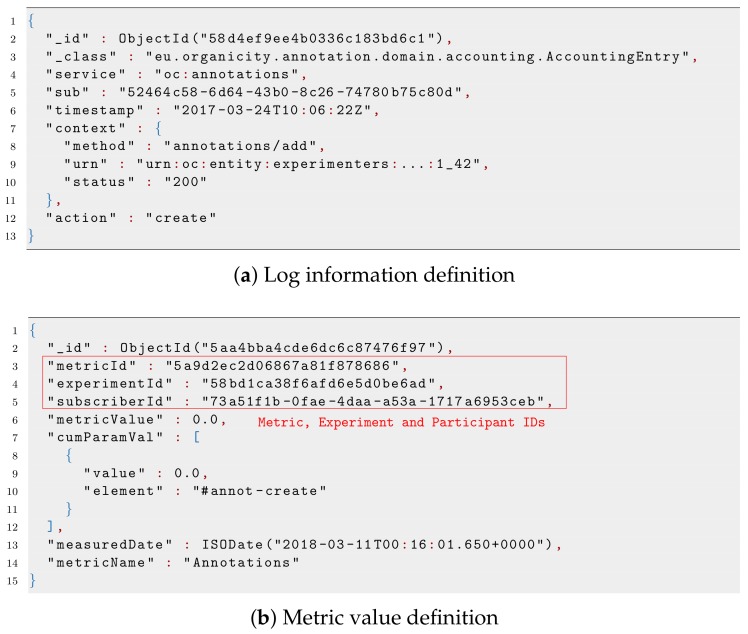
Illustrative examples of the log information and value obtained by one metric, for one participant.

From an implementation perspective, all information related to user engagement is stored in a non-relational database, in which different collections are created, as shown in Figure 5. First, participants and metrics are stored in the same database and separate collections. Then, in a dedicated database, called logs, we store the log entries for the different OrganiCity components, which are afterwards used to calculate the metric value, as we will describe in Section 5. This information indicates actions performed by OC users through the different OC components, and they are stored as depicted in Figure 6a. Two attributes are important in those documents: *sub* and *urn*. The former specifies the identifier of the user that performed the action, while the latter is a structured array with the pattern *urn:oc:entity:experimenters:[ExperimenterID]:[ExperimentID]:[AssetID]* and defines the particular data asset over which the action was performed.

From the log information and metric definition, the metric values can be calculated for each pair participant/experiment, as shown in Figure 5. A script running in the background periodically retrieves the information from the different collections and calculates new values for all metrics. The script uses, as input values, the participant lists and metric definitions of each experiment, as well as the number of interactions carried out by participants, which are registered in the logs database. As output, the script provides the metric values, which are stored in the metric values collection.

The resulting metric value is stored as shown in Figure 6b. The metric identifier to which this value instance belongs is included in the *metricId* field. As can be seen, the metric value also includes identifiers of the experiment and participant, in the *experimentId* and *subscriberId* keys, respectively.

## 5. Engagement Metric

As we have seen before, the citizen engagement is based on two main strategies. On the one hand, communities are used to identify specific niches of potential users and to broaden the experiment’s audience. This also allows experimenters to collect a large amount of data, as well as to use the experiments in stressed environments in order to improve their functionalities. On the other hand, once participants are enrolled in experiments, and it is necessary to define mechanisms to measure their activity level in the experiment, as well as their degree of interaction with the OC platform. Based on the level of activity, the experimenter can define incentive and reward strategies with the aim of encouraging participants to be more active during the experimentation process.

In this sense, the appropriate definition of the engagement metric plays a fundamental role in the overall engagement methodology. However, it is unlikely that a common metric definition can properly tackle the experiment needs due to both the heterogeneity of urban experiments and their different development stages. For that reason, in the OC framework, we opted to provide a common concept of a metric that can be tailored and modified over time according to the experiment’s needs.

When an experiment is executed, it makes use of different components of the platform in order to fulfill the tasks for which it was designed. This interaction is recorded by the corresponding component according to a well-defined format. In this way, a new log entry is created each time the participant uses an element of the platform (for instance, create or read data assets or annotations). Based on this information, it is possible to calculate the level of activity that each participant has carried out within the experiment. We must bear in mind that the information logged only contains data of the components used by the participants. Moreover, the platform implements mechanisms to anonymize the identity of participants so that experimenters cannot identify them.

The level of activity of each participant can produce different ranges of values depending on the frequency of interaction with the platform. For example, at a certain moment, a participant could have created 200 data assets and read only 15, while another one could have created only 40 data assets and read 500. Therefore, to make a fair comparison of the engagement level of both participants, it is necessary to define an evaluation scheme that enables including different types of activities with distinct ranges of values. For this purpose, the engagement metricconcept is defined by using the mathematical model called the utility function. This model enables the experimenter to measure the utility generated by the participant while making use of the platform through an experiment. For example, if a participant creates very few data assets, this would mean a small utility to the experimenter.

In concrete terms, the *Engagement Metric* is defined as a weighted summation of utility functions, as shown in Equation (1):

(1) $$M=\sum_{i=1}^{N}\omega_{i}\cdot f_{i}\left(\rho_{i}\right)$$

where $\omega_{i}$ and $\rho_{i}$ are the relative weight and current value of the parameter *i*, respectively. Each of the *N* parameters represents a measurable element of the platform, which is modeled by a specific utility function $f_{i}$. So far, the following parameters have been defined within the *Engagement Monitoring*:

- $\#discov_{\mathrm{read}}$: number of data assets obtained from the platform.
- $\#discov_{\mathrm{create}}$: number of data assets created and stored on the platform.
- $\#annot_{\mathrm{read}}$: number of data annotations obtained from the platform.
- $\#annot_{\mathrm{create}}$: number of data annotations created and stored on the platform.

When the experimenter includes several utility functions in the metric, the $\omega_{i}$ value can be used to provide a greater or lesser relative importance to each one.

In order to make comparisons between different metrics, we must ensure that the overall metric value is always bounded between zero and one. For this purpose, two conditions must be fulfilled:

- The utility functions are required to be ever-growing ones and unary bounded, $0\leq f_{i}\left(\rho_{i}\right)\leq 1$.
- The sum of all weights must be equal to 1.0, $\sum_{i}\omega_{i}=1$.

Furthermore, we must ensure that the values generated by the utility functions are actually within an admissible range to avoid an imbalance in Equation (1). In this sense, it is necessary to specify the maximum value each involved parameter can reach; this is the maximum of the utility function. In practice, this can be rather unpredictable; thus, instead of defining the maximum parameter value, experimenters define the value for which the utility will take its maximum (i.e., target value). This way, only parameter values that are within the range $\left[0,max_{i}\right]$ will be taken into account for the calculation of the utility, while the utility function will be one otherwise ($f_{i}=1\;\forall \;\rho_{i}\geq max_{i}$). It is worth noting that an experimenter can create several metrics for the same experiment in order to evaluate it from different viewpoints.

**Figure 7:**
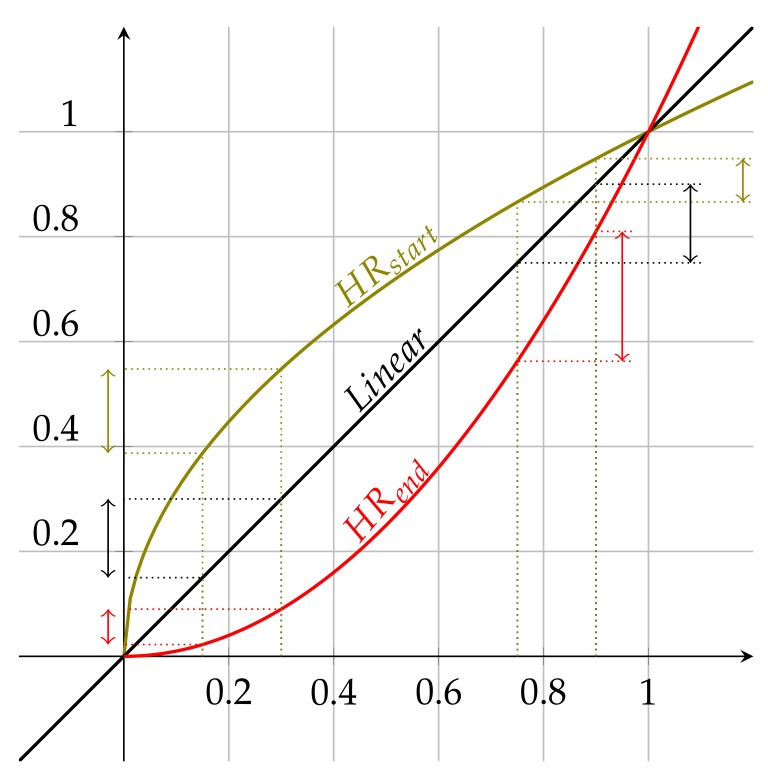
Example of user-defined utility functions. The graph shows the three function shapes currently available within OrganiCity.

Concretely, Figure 7 depicts the utility functions currently implemented to support the *Engagement Monitoring* system. For each utility function, the *X*-axis corresponds to the normalized value of the parameter to be modeled. The normalized value is calculated as a ratio of the actual value and the maximum one for which the function reaches its maximum value ($\rho_{i}/max_{i}$). For example, let us assume that an experimenter has decided to use the $\#annot_{\mathrm{create}}$ (number of annotations created) parameter within a metric, and it is established that the maximum value corresponds to 500 new annotations. If you want to evaluate the utility generated for 150 annotations, then you would have to use the normalized parameter value 150/500=0.3. The value corresponding to the *Y*-axis will depend on the utility function that is used. As can be seen in Figure 7, we use utility functions that take the unary value for the unary input, so the *X*- and *Y*-axes are unary bounded.

In particular, the functions that are used to model the utility of each metric parameter are the following:

- *HRstart* (high rate start), which is based on the $f\left(x\right)=\sqrt{(}x)$ function
- *HRend* (high rate end), which is based on the $f\left(x\right)=x^{2}$ function
- *Linear,* which is based on the linear function f(x)=x

We have chosen these functions to model the utility due to their different properties. The *Linear* function is the simplest one, and it provides the same increase in the utility value for any increase in the parameter value; see Figure 7. On the contrary, the *HRstart* and *HRend* functions show very interesting features. For increments of the parameter value in the lower areas of the *X*-axis, *HRstart* provides a greater increase in utility compared to *HRend*, as shown in Figure 7. However, if you look at the higher areas of the *X*-axis, *HRend* offers a greater increase in utility compared to *HRstart*. Depending on the scenario in which the experimenter is involved, it may be convenient to model the utility with one or another function. Below, we provide some examples to illustrate the criteria to choose a certain utility function.

### Examples of Metric Creation

Let us imagine that an experimenter wants to know which participants feed into or consume more data from the OC platform. For this particular use case, the experimenter defines a metric using two parameters: the number of assets that each participant has created and the number of assets consumed from the platform.

- $\rho_{1}$ = Number of assets created ($\#discov_{\mathrm{create}}$)
- $\rho_{2}$ = Number of assets consumed ($\#discov_{\mathrm{read}}$)

As part of the design criteria, the experimenter also decides to use linear functions (y=x) to model the utility of each parameter. In this way, an increase in the number of created or consumed assets will provide a proportional increment in the utility.

- $f_{1}\left(\rho_{1}\right)$ = Linear function
- $f_{2}\left(\rho_{2}\right)$ = Linear function

Due to the characteristics of the experiment, it is also decided to use different weights for both metrics, in such a way that the number of assets consumed is more relevant than those created. To do so, the experimenter assigns a higher weight to the function corresponding to the second parameter.

- $\omega_{1}=0.4$
- $\omega_{2}=0.6$

Finally, taking into account the aforementioned design criteria, the metric, *M*, would be defined as shown in Equation (1):

$$\begin{array}{ccc} M & = & \omega_{1}\cdot f_{1}\left(\rho_{1}\right)+\omega_{2}\cdot f_{2}\left(\rho_{2}\right)\\ M & = & 0.4\cdot Linear\left(\#discov_{\mathrm{create}}\right)+0.6\cdot Linear\left(\#discov_{\mathrm{read}}\right) \end{array}$$

Let us now imagine that the experimenter does not want to give more relevance to any of the utility functions corresponding to the parameters; i.e., $\omega_{1}=\omega_{2}=0.5$. Instead, the experimenter decides to use different functions so that an increase in the number of assets created or consumed will generate different increases in the utility function.

In particular, the experimenter intends to encourage the participant to consume more assets, while not showing special interest in the creation of assets. For the case of the consumed assets, this means that a greater increase in the utility should be produced when the quantity of consumed assets is increasing. To accomplish this requirement, the *HRend* function would have to be used. On the other hand, in the case of created assets, the increase in the utility must be the same for any value of asset created, so the *linear* function would have to be used.

- $f_{1}\left(\rho_{1}\right)$ = Linear function
- $f_{2}\left(\rho_{2}\right)$ = HRend function

Therefore, the new metric would be defined as follows:

(2) $$M=0.5\cdot LinearFunction\left(\#discov_{\mathrm{create}}\right)+0.5\cdot HRendFunction\left(\#discov_{\mathrm{read}}\right)$$

## 6. Urban Services Validation

Within the OrganiCity project, two open calls were launched to validate the developed platform externally. The first one, which took place in 2016/2017, aimed to analyze the initial version of the facility and to provide feedback in order to improve and extend it. On the other hand, the second open call, which was launched in 2017/2018, focused on assessing the capabilities of the facility to accelerate the creation of novel urban services.

During the first open call, the experimentation management ecosystem was released in its first version, while it was further extended in the second open call by including the CP and the whole engagement functionality presented in this paper. In addition, experiments executed during the open calls brought about a set of reports in which experimenters explained the progress of their urban solution and the usage of the platform.

Among other objectives, these reports served to analyze the sustainability of the OC framework, which depends on two aspects: the sustainability of the services developed within OC and the level of integration, or dependency, of experiments with the OC platform. As for the experiment’s sustainability, we have made use of the information provided by the experiment teams in their final reports, where business plans and support are reported. On the other hand, the evaluation of the technical integration is based on factual information provided by the OrganiCity platform itself.

In the following, we summarize the second open call experimenters’ feedback, focusing on the acceptance of the OC services in general, and the experimentation management in particular, and the final outcome of the experiments. Then, we will conclude the section describing the potential use of the OrganiCity user engagement functionalities, both for industry and citizens.

### 6.1. Adoption of the OrganiCity Platform

**Figure 8:**
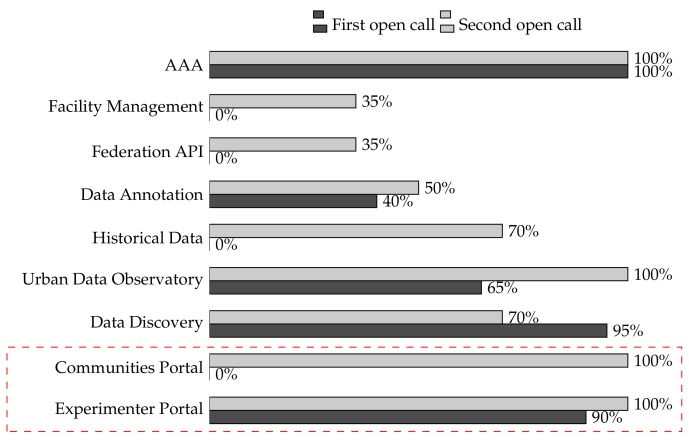
OrganiCity service adoption level during the first and second open calls.

Figure 8 shows, for the main OC services, the ratio of experiments that used them in the first and second open calls.

Among the services enumerated in Figure 8, we can distinguish three groups. The first one embraces services dedicated to the framework management (AAA, facility managements, and federation API). The second group comprises data-related services, either as APIs or through the UDO, such as the data annotation, discovery, and historical data. Finally, the experimenter portal and communities portal represent the experimentation management services.

In general, we can observe a higher adoption level of the OC services during the second open call. The only exception is the data discovery service, most likely due to the fact that the second open call services were rather focused on specific urban challenges, so that they exploited very concrete data assets. In addition, the evaluation indicates that both the federation API and facility management functionalities were only used in the second open call. In this case, the reason is the federation of new cities within the OC platform.

Concerning the experimentation management services, Figure 8 indicates that all experiments made use of both the EP and CP during the second open call. It is worth noting that the CP was not released until the second open call, after receiving the feedback from experiments developed in the first open call. The statistics used in this evaluation only concern experimenters, and for this reason, the PP is not included in Figure 8 since it was only used by the participants.

### 6.2. Improving the Maturity Level of Future Urban Services

After analyzing to what extent experiments exploited the different functionalities brought about by the OC platform, here, we study how the platform helped to increase the technical readiness level (TRL) of urban services developed within the experiments. To this end, experimenters were explicitly asked about this topic in their reports.

**Figure 9:**
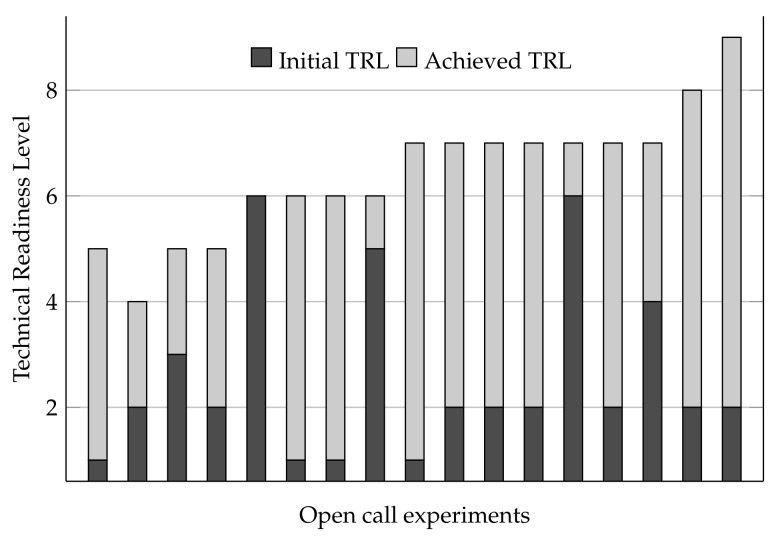
Technical readiness evolution of the experiments during the OrganiCity second open call according to experimenters’ feedback. Concrete experiments’ names are omitted.

Figure 9 depicts the evolution of services’ TRL before and after the second open call. It is worth noting that this parameter was not analyzed in the first open call since, as mentioned before, it was not its objective. In addition, we point out that the results shown in Figure 9 were obtained from the experiments’ reports, so they only represent the perception and opinion of the experimenters.

As can be seen, almost all experiments experienced a notable TRL increase during the experimentation period. In addition, we can also observe that the higher increment corresponds to those experiments that started with the lowest TRL. On the other hand, experiments that started with a relatively high TRL did not evolve much. In addition, although some experiments claimed to have reached TRL 8 or 9 (fully qualified and operational systems, respectively), we attribute this overestimation to a lack of understanding of such levels, since neither the experimentation periods, nor the OC platform were conceived of for that purpose.

Altogether, Figure 9 reveals that most of the experiments reached a maturity level close to the commercial or industrial state. In light of these results, we can conclude that the OrganiCity platform has served to demonstrate innovative ideas in relevant scenario and as an effective accelerator to develop experimental services, leading such services to the prototype state (TRL 7).

### 6.3. Impact on Industrial Stakeholders

Taking into account the holistic vision promoted by OrganiCity, the different functionalities implemented there have been designed with multiple objectives, taking into account the needs of the stakeholders involved. In particular, and considering the above results, it is possible to describe how the engagement monitoring functionalities can be exploited by industrial stakeholders.

The particular needs and objectives of private companies will dictate the adoption of OrganiCity by private companies. On the one hand, large industries could consider adopting the platform as a whole, taking advantage of the features offered by this EaaS initiative to include new functionalities in their portfolios, and gain valuable experience in a field that could become increasingly important in the near future, as cities become aware of the benefits that can be obtained through this type of approach.

On the other hand, small and medium enterprises (SMEs) should consider the possibility of developing specific components or even exposing new hardware devices that in turn can become part of the OrganiCity platform, generating themselves the data that can be exploited later not only by them, but also by other experimenters who might show interest in having these data. These small companies, through a sensible use of this platform, can take a step forward in innovation, competitiveness, and growth. Among the potential advantages to be exploited is the capacity that the facility provides to reduce the cost of entering the market, taking advantage of the fact that most of the platform they would require is already built, eliminating development and installation costs. In this way, SMEs focus on their basic services and avoid the cumbersome development of certain generic elements. This opens the door to new markets, allowing them to participate in an EaaS platform that would place them in a leading position in this environment.

In all cases, the ability to evaluate how the participants of the experience interact with the experimental services makes it possible to define various actions and measures to be taken to satisfy the wishes of potential customers. The world is heading, from a technological and market point of view, towards a Fourth Industrial Revolution, where a company that focuses on the strict manufacturing of what the customer wants, while creating high quality and personalized products and/or services, will incur lower costs and will reduce the waste and inefficiency of current mass production processes, thus diminishing the environmental impact and contributing to sustainability. For example, an SME could take advantage of the commitment metrics generated by a given service placed on the market to better understand its strengths and weaknesses and refine and adapt that service to obtain a valuable product that provides revenue to the company. As an adaptation, consideration should even be given to the possibility of giving certain incentives to customers who actually use that service, something that could be inferred from the above-mentioned metrics.

In addition, such actions and initiatives represent a step forward in the current process of traditional consumers becoming prosumers, as they become responsible for the product or service they are going to use, acting as co-designers and actors, while obtaining detailed information about consumer preferences and requirements will help the industry to create faster and shorter innovation cycles, where the challenge is to combine shorter time-to-market with the need for longer time-on-market.

### 6.4. Impact on Citizens

Nowadays, citizens are not just a passive source of information. Through the democratization of public services that EaaS represents, they are also empowered to be the ones that come up with the answers and solutions to the challenges they experience in their everyday lives. One barrier however is the technical aspect of executing this type of experiment. Not many citizens have the technical skills to use the OrganiCity facility at its current maturity stage, even though the tools that are provided are intended to lower this barrier. In order to overcome this potential usage limitation, OC users have been coupled during the experimentation. For instance, those who are strong in, for example, co-creation have been connected with citizens that are strong in technical knowledge. In this sense, the community concept and its implementation as a community portal have helped to achieve these goals.

On the other hand, engaging and giving support to citizens and communities that could be potential experimenters requires many resources based on the experiences in OrganiCity so far, since guidance and hand holding are needed in every single phase. Therefore, taking the potential experimenters by the hand from the very beginning can also open the door to EaaS for citizens who are not typical experimenters (those who are usually active in urbanism and the Internet of Things). In this sense, the experimentation management ecosystem developed around the key concept of the experiment has made it easier to overcome this barrier, helping to get a higher conversion rate of citizens becoming experimenters or participating in experiments, which is fundamental to support any business model.

## 7. Conclusions

The adoption of ICT and IoT solutions under the smart city paradigm is making the urban environment an even more changing and challenging scenario. In this context, it is necessary to develop tools that can accelerate the adoption and deployment of urban solutions in an agile way, in order to meet the ever-changing user needs.

The OrganiCity project has proposed a novel EaaS reference architecture and implementation devoted to simplifying the deployment and development of novel urban services so that they can be validated at a small scale by means of experiments. On top of that, the experimentation flow fostered by OrganiCity follows a co-creative approach that will ultimately lead to services better suited to the users’ needs. In this sense, it is of utter importance that citizens become engaged during the whole experiment lifecycle.

In this work, we have presented the engagement monitoring framework, which provides mechanisms to measure the engagement level of citizens. On the one hand, the framework allows grouping of people sharing common interests, by means of the creation of communities. This way, experimenters can better address their potential users and enroll them in their experiments.

Once citizens take part in experiments, the presented framework permits the ad-hoc creation of engagement metrics according to the experiment particularities. In particular, experimenters can define a metric as a multi-criteria utility function considering different types of interactions of the experiment participants with the OC platform. Afterwards, it is possible to monitor the evolution of citizens’ engagement according to the defined metrics. Eventually, experimenters can decide to perform modifications on their services or to undertake incentivization campaigns.

The engagement monitoring services were released as part of the advanced experiment features deployed in the 2nd OrganiCity Open Call. According to the reported feedback, the presented functionalities have been largely adopted by experimenters and have helped to accelerate the maturity of experimental services. Altogether, the engagement monitoring framework is a valuable asset for all the stakeholders involved in the creation, development, and exploitation of urban services, since it brings about the possibility to analyze the behavior of their potential users upon both service modifications and incentivization initiatives.

## Abbreviations

EaaS	experimentation-as-a-service
EM	experimentation management
IoT	Internet of Things
AAA	authentication, authorization, and accounting
OC	OrganiCity
API	application programming interface
EP	experimenter portal
CP	communities portal
PP	participant portal
ICT	information and communication technologies
UDO	Urban Data Observatory
TRL	technical readiness level
SME	small and medium enterprise
JSON	JavaScript Object Notation
